# Underreporting of oral cleft cases: a descriptive study, western Paraná, 2015-2019

**DOI:** 10.1590/S2237-96222026v35e20240716.en

**Published:** 2026-03-20

**Authors:** Isabelle Leticia Bender de Souza, Andressa Brum, Aline Nardelli, Mariângela Monteiro de Melo Baltazar, Fabiana Gonçalves de Oliveira Azevedo Matos, Luciana Paula Grégio d’Arce

**Affiliations:** 1 Universidade Estadual do Oeste do Paraná, Cascavel, PR, Brazil

**Keywords:** Congenital Anomalies, Cleft Palate, Cleft Lip, Health Service, Epidemiology, Anomalías Congénitas, Fisura del Paladar, Labio Leporino, Servicio de Salud, Epidemiología

## Abstract

**Objectives:**

To verify whether there was underreporting of oral cleft cases on the Live Birth Information System (*Sistema de Informações Sobre Nascidos Vivos*, SINASC) in the 10th Paraná Health Region, analyzing agreement between SINASC data and those recorded at a referral center in the same health region, as well as to define average prevalence in the period 2015-2019.

**Methods:**

This is a descriptive and retrospective epidemiological study. Data were obtained from both databases according to International Classification of Diseases (10th revision) codes. The ratio between cases and live births in the same year and location was used as a frequency measure.

**Results:**

We analyzed 121 cases, 44 based on SINASC data and 77 based on referral center data. According to SINASC, the prevalence rate was 8.75 cases per 10,000 inhabitants, while according to the referral center it was 16.87 cases per 10,000 inhabitants, indicating 42.86% underreporting on SINASC. Regarding the analysis of oral cleft classification, 68.19% of cases had their cleft type determined on the SINASC.

**Conclusion:**

The study demonstrated that, in the 10th Paraná Health Region, there was underreporting on SINASC of people born with oral clefts between 2015 and 2019. This could perhaps have been resolved if the data on the live birth certificate had been edited after birth when the cleft was detected.

## Resumen

**Objetivos:**

Verificar si existía subregistro de casos de fisura oral en el Sistema de Información sobre Nacidos Vivos (*Sistema de Informações Sobre Nascidos Vivos*, SINASC) en la 10ª Región Sanitaria de Paraná, analizando la concordancia de las cifras con las registradas en un Centro de Referencia de la misma región, así como definir la prevalencia promedio en el período 2015-2019.

**Métodos:**

Estudio epidemiológico, descriptivo y retrospectivo. Los datos se obtuvieron de ambas bases de datos de acuerdo con los códigos de la 10ª revisión de la Clasificación Internacional de Enfermedades. La razón entre casos y nacidos vivos en el mismo año y ubicación se utilizó como medida de frecuencia.

**Resultados:**

Se analizaron 121 casos, 44 con datos del SINASC y 77 con datos del Centro de Referencia. Según el SINASC, la prevalencia fue de 8,75 casos por 10.000 habitantes. Según el Centro de Referencia, se encontró una prevalencia de 16,87 casos por cada 10.000 habitantes, lo que indica un subregistro del 42,86%. En cuanto al análisis de la clasificación de las fisuras orales, el 68,19% de los casos tuvieron su tipo de fisura determinado en el SINASC.

**Conclusión:**

El estudio demostró que, en la 10ª Región Sanitaria de Paraná, hubo un subregistro en el SINASC de personas nacidas con fisura oral entre 2015 y 2019. Esto podría haberse solucionado si los datos del certificado de nacido vivo se hubieran editado después del nacimiento, cuando se detectó la fisura.

**Ethical aspects**

This research respected ethical principles, having obtained the following approval data:

Research ethics committee: Universidade Estadual do Oeste do Paraná

Opinion number: 4,250,143

Approval date: 1/9/2020

Certificate of submission for ethical appraisal: 36452320.0.0000.0107

Informed consent form: Not applicable.

## Introduction 

Oral clefts are the most frequent congenital anomalies in the population among craniofacial anomalies (1-3). Oral cleft etiology is related to hereditary factors, estimated at between 25% and 30% in 2023, which may be related to Mendelian inheritance or chromosomal alterations (4), and to a range of multifactorial environmental factors. These factors include nutritional deficiencies, alcoholism, lifestyle and parental exposure to chemicals such as pesticides, corresponding to between 70% and 80% of occurrences in 2019 (5,6). Seasonality, ethnic group, parental age, birth weight and social class have also been related oral cleft etiology (1,7-9).

Prevalence of live births with oral clefts in Brazil was approximately 5.2 per 10,000 births in 2022 (10). In 2025, the Southern region continued to stand out with the highest prevalence of clefts in the country (5,10,11). Craniofacial malformations, such as oral clefts, show an upward trend and are of great epidemiological importance, in addition to social and economic impacts for the individual, their family and society in general (5,12). Treatment requires an integrated approach with a highly specialized multidisciplinary team (13).

Due to the need for monitoring health data, the Brazilian Unified Health System has implemented facilities such as its Department of Information Technology (*Departamento de Informática do Sistema Único de Saúde*, DATASUS), with the aim of systematizing and disseminating information through electronic data transfer. In addition, DATASUS is the place where operational and epidemiological indicator data are produced, which underpin public health policies (14,15).

These include the Live Birth Information System (*Sistema de Informações Sobre Nascidos Vivos*, SINASC), which uses International Classification of Diseases - 10^th^ revision (ICD-10) codes to classify the various types of clefts. These correspond to ICD-10 Q35 (cleft palate), Q36 (cleft lip) and Q37 (cleft lip and palate) (16).

When a baby is born, the health professional in charge has to complete the Live Birth Certificate, the basic document for SINASC data entry. It contains general information about the newborn, and its item 6 reports whether any congenital anomaly was detected, which must be described in item 41. The corresponding ICD-10 code is entered in this field, including oral clefts (17).

The objective of this study was to verify whether there was underreporting of oral cleft cases among newborns between 2015 and 2019 in the 10^th^ Health Region of the State of Paraná, based on comparison between data recorded on SINASC and those recorded at an oral cleft referral center located in the same region. Furthermore, it aimed to determine the prevalence of oral clefts in this population. 

## Methods 

### Design 

This is a descriptive and retrospective epidemiological study conducted between March and July 2024. We analyzed paper and electronic medical records from the Craniofacial Anomaly Care and Research Center (*Centro de Atenção e Pesquisa em Anomalias Craniofaciais*), located at the University Hospital of Western Paraná (*Hospital Universitário do Oeste do Paraná*), and SINASC data, covering the period from 2015 to 2019.

### Setting 

The study used data on the 10^th^ Paraná Health Region (Cascavel region, Paraná), obtained from both SINASC and the referral center.

The referral center has existed since 2013, being officially accredited by the Brazilian Unified Health System in 2018 for treatment of people with highly complex cleft lip and palate conditions. It offers specialized treatment for people with craniofacial anomalies, especially cleft lip and palate, mainly from the western and southwestern regions of the state of Paraná, as well as other states such as Santa Catarina and Mato Grosso do Sul, and neighboring countries such as Paraguay and Venezuela.

### Study size 

The 10^th^ Health Region covers 25 municipalities in the western region of the state of Paraná. Its population was estimated at 510,000 inhabitants in 2025.

### Data source

All medical records from the referral center relating to the 10^th^ Health Region and births recorded on SINASC in the same Health Region between 2015 and 2019 were taken into account, taking the mother’s municipality as the municipality of residence, this being a criterion used by SINASC.

### Data collection

The following ICD-10 codes were used to obtain data from SINASC:

- Q35 for types of cleft palate (Q35.1 Cleft hard palate; Q35.3 Cleft soft palate; Q35.5 Cleft hard palate with cleft soft palate; Q35.7 Cleft uvula; and Q35.9 Cleft palate, unspecified);
- Q36 for cleft lip (Q36.0 Cleft lip, bilateral; Q36.1 Cleft lip, median; and Q36.9 Cleft lip, unilateral); and
- Q37 Cleft palate with cleft lip (Q37.0 Cleft hard palate with bilateral cleft lip; Q37.1 Cleft hard palate with unilateral cleft lip; Q37.2 Cleft soft palate with bilateral cleft lip; Q37.3 Cleft soft palate with unilateral cleft lip; Q37.4 Cleft hard and soft palate with bilateral cleft lip; Q37.5 Cleft hard and soft palate with unilateral cleft lip; Q37.8 Unspecified cleft palate with bilateral cleft lip; and Q37.9 Unspecified cleft palate with unilateral cleft lip).

The data were collected manually from SINASC, according to the ICD-10 codes, and tabulated using Microsoft Excel.

The referral center data were obtained by analyzing the medical records of people born during the analysis period, based on the description of the type of cleft made by the Center’s multidisciplinary team.

### Statistical methods

In order to assess SINASC sensitivity in identifying oral cleft cases, a comparison was made with the referral center data. Cases registered simultaneously on both systems were considered true positives, and cases registered exclusively by the referral center were considered false negatives. Sensitivity was calculated as the ratio between the number of cases registered by both systems and the sum of true positives and false negatives.

Prevalence of oral clefts in newborns, both from the referral center and from SINASC, was calculated by the ratio between the number of live births with clefts and the total number of live births held on SINASC, in both the same year and the same location, multiplied by 10,000. Assessment of the type of cleft was based on SINASC data, which categorizes them according to ICD-10, where cleft palates were designated as Q35; cleft lips as Q36; and cleft lips and palates as Q37. The differences between the datasets were calculated, and the relevant percentage rates were established. Clefts without a specified ICD-10 code were not included in the analysis by cleft type.

In order to assess whether there was statistically significant difference between the results of the databases, the variables were analyzed for normality (Shapiro-Wilk test). If the normality assumption was met, comparisons between the two databases were performed using the one-tailed t-test for dependent samples, and if not, the non-parametric Wilcoxon test was used, with a 5% significance level. The maps were generated using the free QGIS software.

## Results 

Analysis of the period in question revealed 44 oral cleft cases recorded on SINASC, while the Referral center reported 77 cases in the same period. SINASC sensitivity was 57.14%, which indicates that the system was able to capture just over half of the cases actually recorded by the Referral center.

**Figure 1:**
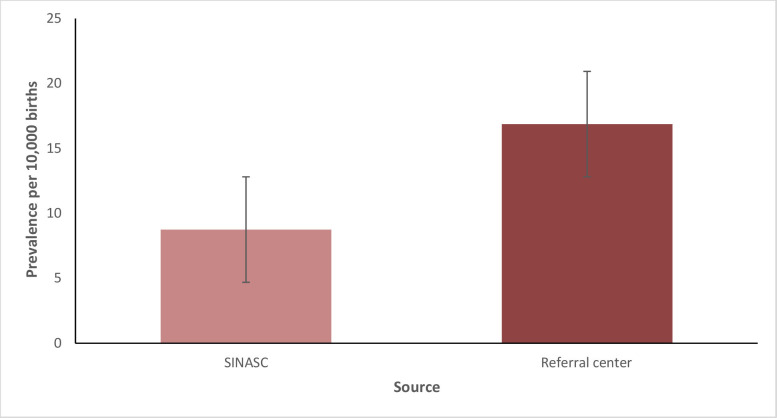
Prevalence per 10,000 births in the 10^th^ Health Region according to the Live Birth Information System (SINASC) and the regional Referral center. Paraná, 2015-2019

Assessment of the SINASC records revealed average prevalence of 8.75 oral cleft cases per 10,000 births between 2015 and 2019, which corresponded to 1 case per 1,143 births. The Referral center data showed prevalence of 16.87 cases per 10,000 births, equivalent to 1 case per 593 births (Figure 1). The underreporting rate was 42.86%. There was a statistically significant difference between the average prevalence rates of the databases analyzed (p-value 0.001).

**Figure 2:**
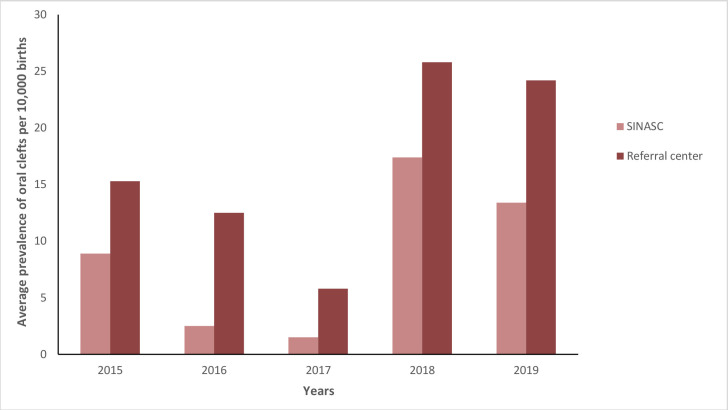
Average annual prevalence of oral clefts per 10,000 births in the 10^th^ Health Region according to the Live Birth Information System (SINASC) and the regional Referral center. Paraná, 2015-2019

According to the SINASC system, average prevalence of oral clefts in the 10^th^ Paraná Health Region in 2015 was 8.9 cases per 10,000 inhabitants; decreasing to 2.5 in 2016 and to 1.5 in 2017; increasing to 17.4 in 2018; and reaching 13.4 in 2019. With regard to the Referral center, the prevalence rate was 15.3 cases per 10,000 births in 2015; decreasing to 12.5 in 2016 and to 5.8 in 2017; and increasing significantly to 25.8 in 2018 and 24.2 in 2019. When comparing the prevalence rates, an increase in oral cleft cases was observed, especially in 2018 and 2019. Prevalence was higher at the Referral center when compared to SINASC in the years mentioned (t=-7.98; p-value 0.001) (Figure 2).

Among male cases, a statistically significant difference was observed between the average prevalence rates of the Referral center compared to SINASC (p-value 0.011). The prevalence rate was 10.34 cases per 10,000 births according to SINASC data, while the Referral center recorded 22.29 cases. Among female cases, there was no statistically significant difference between the databases (p-value 0.421), with averages of 6.70 cases per 10,000 births on SINASC and 10.46 at the Referral center.

**Figure 3:**
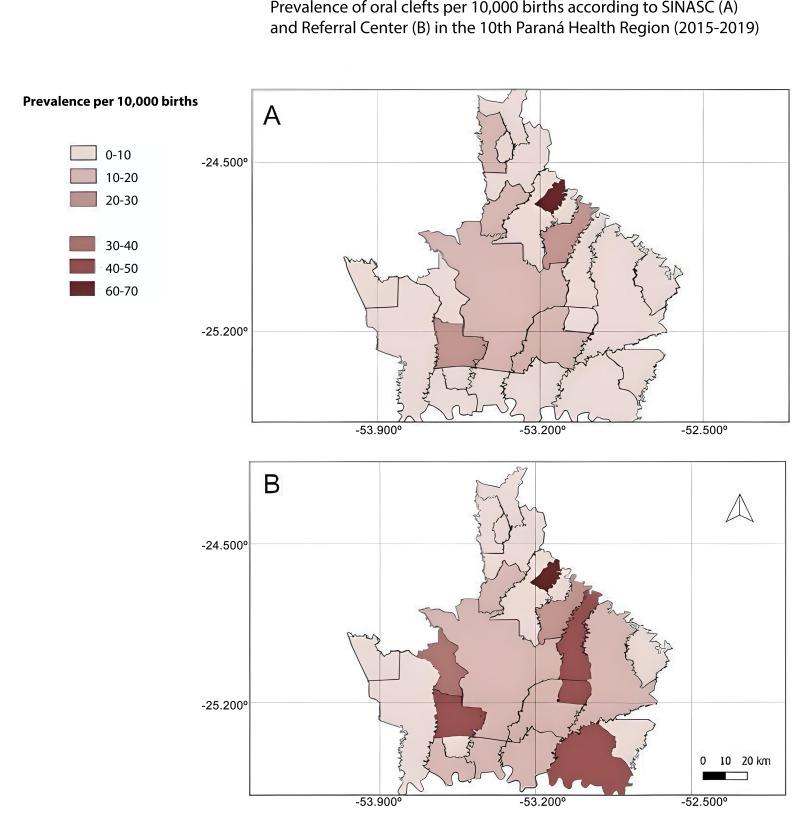
Prevalence of oral clefts per 10,000 births between 2015 and 2019 by municipality in the 10^th^ Paraná Health Region, according to data from: (A) Live Birth Information System; (B) regional Referral center, 2015-2019

According to SINASC data, 1 municipality presented a prevalence rate between 60 and 70 cases per 10,000 births, 3 municipalities registered a prevalence rate between 20 and 30 cases, and 4 municipalities had a prevalence rate between 10 and 20 cases (Figure 3A). Eighteen municipalities presented a prevalence rate of less than 10 cases per 10,000 inhabitants (Figure 3A).

According to Referral center data, 1 municipality recorded a prevalence rate between 60 and 70 cases per 10,000 births, 4 municipalities presented a prevalence rate between 40 and 50 cases, 1 municipality had a prevalence rate between 30 and 40 cases, 2 municipalities recorded a prevalence rate between 20 and 30 cases, and 6 municipalities presented a prevalence rate between 10 and 20 cases (Figure 3B). Eleven municipalities presented a prevalence rate below 10 cases per 10,000 births (Figure 3B).

Cleft cases were broadly classified as lip, palate, and both lip and palate. Analyzing SINASC data without distinguishing by sex, cleft palates accounted for 8 cases (18.9%), being the least frequent, followed by cleft lips with 9 cases (20.5%) and lip and clefts with 13 cases (29.6%). Fourteen cleft occurrences registered on SINASC did not have an ICD code specified on the system (31.8%). The type of cleft was determined on the system in 68.2% of cases.

**Figure 4:**
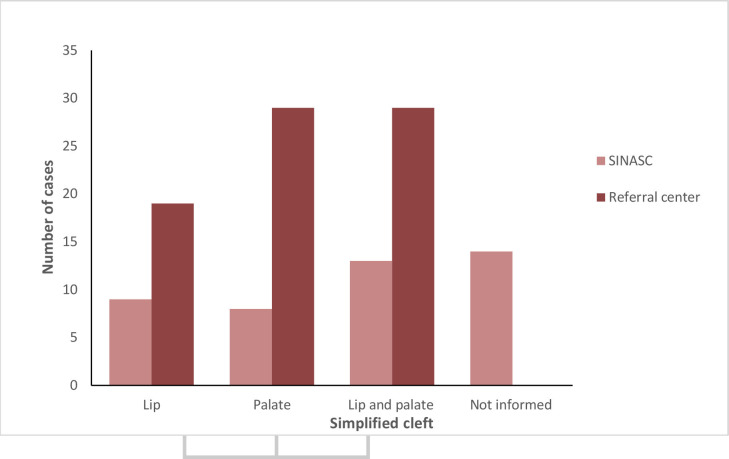
Number of cases by simplified type of cleft recorded on the Live Birth Information System (SINASC) and at 10^th^ Health Region Referral center. Paraná, 2015-2019

The Referral center data indicated that both cleft palate and cleft lip and palate comprised 29 cases (37.7%) out of the total. Cleft lip totaled 19 cases (24.7%) (Figure 4). Among the cleft lip cases recorded by the Referral center, 52.7% were not specified on SINASC. Regarding cleft lip and palate, this number was 55.2%, with the greatest discrepancy observed for cleft palate, which reached 72.4%.

## Discussion 

This study analyzed SINASC data on oral clefts, comparing them with those from a Referral center. A higher prevalence of oral clefts was found in the 10^th^ Health Region compared to the average for the state of Paraná, and underreporting of clefts on SINASC was identified.

Prevalence of oral cleft cases according to SINASC records and Referral center records, 8.75 and 16.87 per 10,000 inhabitants respectively, proved to be higher than in previous national studies. In the state of São Paulo, between 1975 and 1994, a prevalence rate of 1.9 cases per 10,000 births was observed (18). Data from 2000 onwards showed higher prevalence rates: 5.8 per 10,000 inhabitants in Rio Grande do Norte in 2017 (19), 5.2 in Minas Gerais in 2022 (10) and 5.1 cases in Paraná in 2019 (5).

The Southern region has the highest rates of oral clefts in Brazil, with a prevalence rate in 2022 of 7.16 cases per 10,000 births (10), 7.9 cases in 2019 (5), and 8.23 ​​cases in 2017 (19). Comparing the prevalence rates in the literature with those described in this study, we found that the 10^th^ Paraná Health Region presented higher prevalence rates both in the data obtained from SINASC and also in those obtained from the Referral center.

The prevalence of oral clefts reported here for the Referral center, for a single health region, was higher than that previously reported for the state of Paraná as a whole (6.8 cases per 10,000 births) (19). Similarly, another study published with data from another center specializing in oral clefts estimated this prevalence rate at 9.9 cases per 10,000 births, this also being higher than the average for Paraná (20).

These higher oral cleft prevalence rates identified in Referral centers highlight the importance of further investigation in order to understand the specific factors contributing to these regional variations in cleft rates. Furthermore, they will contribute to more effective prevention and intervention strategies for oral cleft cases.

Regarding the prevalence data for oral clefts found in the literature, they differ from each other due to several factors, including multifactorial etiologies and difficulty in obtaining and characterizing samples accurately. These factors partly explain the inconsistencies between public databases and those of referral centers (21,22).

SINASC is fed with data from live birth certificates, aiming to collect information about pregnant women and newborns in order to outline an epidemiological profile. Although the certificates include fields for information on congenital anomalies, underreporting frequently occurs due to various reasons, such as lack of diagnosis at birth and lack of awareness of the need for reporting on the part of the health professionals who fill out the document. The limited timeframe for data entry to the system compromises data quality and hinders the planning of actions aimed at neonatal care and specialized care.

Oral cleft cases, when not reported in the live birth certificate, will be included later in public databases (as relative numbers) after the start of care provided in the public health system (usually in specialized referral centers). Even with a time lag, these centers play a significant role in the collection of epidemiological data, showing greater accuracy in determining the total number of cases.

This study identified 43% underreporting, with cleft palates being the most underreported, accounting for 72%. In the state of Rio de Janeiro, 45% underreporting was identified on live birth certificates regarding the presence of oral clefts, whereby cleft palates were the most underreported, accounting for 65% (23).

The finding of such underreporting in this study is also aligned with previous findings reported in the literature (20,22,24). The complexity in obtaining and integrating data reinforces the need for improvements in reporting and recording systems for a more accurate assessment of cleft prevalence.

The predominance of cleft palate underreporting can be explained by the difficulty in visualizing clefts immediately after birth, often requiring days or even months before being identified. An additional fact of concern is that only 68% of cases have the type of cleft determined on the SINASC. This information is not only crucial for justifying the allocation of funds, but is also fundamental for defining effective public policies in the Health Region. Even though the Referral center analyzed in this study was only incorporated into the Brazilian Unified Health System network in 2018 (despite being operational since 2013), it is evident that it has improved the collection of new data, as well as retroactive data.

The discrepancy between the databases highlights the need for improvement in the collection of data that is input to SINASC. One strategy would be to provide better training for the health professionals responsible for completing the live birth certificate, given the crucial role they play in defining epidemiological profiles and implementing specialized and referral centers for the integrative treatment of these individuals, in addition to planning budget allocations that are consistent with reality.

Another viable strategy to increase data reliability would be the possibility of later input or modification of data on the SINASC platform. This flexible approach could offer a more dynamic way to update and correct information, thus contributing to a more accurate and useful database for the formulation of effective health policies.

The 10^th^ Paraná Health Region presented higher prevalence of cleft lip and palate cases than previously identified for the state of Paraná, the Southern region of Brazil, and even the national prevalence rate. Although this study focuses on a specific Health Region, we believe it reflects a broader reality of underreporting of public health data at the national level. This problem requires more comprehensive discussion in order to identify ways to minimize this discrepancy and mitigate its effects. Addressing underreporting is fundamental to ensuring an accurate understanding of oral cleft incidence and, consequently, developing effective intervention strategies.

## Data Availability

The database is available at: http://doi.org/10.17632/n9pvg96h55.1.

## References

[1] Setó-Salvia N, Stanier P (2014). Genetics of cleft lip and/or cleft palate: association with other common anomalies.. Eur J Med Genet.

[2] Mossey P, Modell B (2012). Epidemiology of oral clefts 2012: an international perspective.. Front Oral Biol.

[3] Pereira AV, Fradinho N, Carmo S, Sousa JM, Rasteiro D, Duarte R (2018). Associated malformations in children with orofacial clefts in Portugal: a 31-year study.. Plast Reconstr Surg Glob Open.

[4] Babai A, Irving M (2023). Orofacial clefts: genetics of cleft lip and palate.. Genes (Basel).

[5] Shibukawa BMC, Rissi GP, Higarashi IH, Oliveira RR (2019). Factors associated with the presence of cleft lip and/or cleft palate in Brazilian newborns.. Revista Brasileira de Saúde Materno Infantil.

[6] Candotto V, Oberti L, Gabrione F, Greco GB, Rossi DS, Romano M (2019). Current concepts on cleft lip and palate etiology.. J Biol Regul Homeost Agents [Internet].

[7] Silva HPV, Arruda TTS, Souza KSC, Bezerra JF, Leite GCP, Brito MEF (2018). Risk factors and comorbidities in Brazilian patients with orofacial clefts.. Braz Oral Res.

[8] Maranhão SC, Sá J, Cangussú MCT, Coletta RD, Reis SRA, Medrado ARAP (2021). Nonsyndromic oral clefts and associated risk factors in the state of Bahia, Brazil.. European Archives of Paediatric Dentistry.

[9] Luiz AF, Rodrigues LPGA, Demarco NR (2022). Influência da sazonalidade na incidência de fissuras labiopalatinas em um centro de referência no Oeste do Paraná.. Research, Society and Development.

[10] Silva RS, Macari S, Santos TR, Werneck MAF, Pinto RS (2022). The panorama of cleft lip and palate live birth in Brazil: follow-up of a 10-year period and inequalities in the health system.. The Cleft Palate Craniofacial Journal.

[11] Xavier LAC, Bezerra JF, Rezende AA, Oliveira RAC, Dalmolin RJS, Amaral VS (2017). Analysis of genome instability biomarkers in children with non-syndromic orofacial clefts.. Mutagenesis [Internet].

[12] Costa ATA, Holanda JKN, Souza LDG, Custódio LLP, Silva MLD, Monteiro DLA (2021). Perfil das internações de crianças por fissuras labiais e/ou palatinas na região Nordeste do Brasil.. Research, Society and Development.

[13] Freitas JAS, Garib DG, Oliveira M, Lauris RCMC, Almeida ALPF, Neves LT (2012). Rehabilitative treatment of cleft lip and palate: experience of the Hospital for Rehabilitation of Craniofacial Anomalies - USP (HRAC-USP) - Part 2: Pediatric Dentistry and Orthodontics.. Journal of Applied Oral Science.

[14] Lima AC, Januário MC, Lima PT, Silva WM (2015 ). DATASUS: o uso dos Sistemas de Informação na Saúde Pública.

[15] Silva PMS, Autran MMM (2019 ). Repositório DATASUS: organização e relevância dos dados abertos em saúde para a vigilância epidemiológica.

[16] Brasil. Ministério da Saúde. (2020 ). Sistema de Informações sobre Nascidos Vivos (Sinasc).

[17] Brasil. Ministério da Saúde. (2022.). Declaração de Nascido Vivo: Manual de instruções para preenchimento [Internet]..

[18] Loffredo LCM, Freitas JAS, Grigolli AAG (1975). Prevalência de fissuras orais de 1975 a 1994.. Rev Saude Publica.

[19] Sousa GFT, Roncalli AG (2017). Orofacial clefts in Brazil and surgical rehabilitation under the Brazilian National Health System.. Braz Oral Res [Internet].

[20] Souza J, Raskin S (2013). Clinical and epidemiological study of orofacial clefts.. J Pediatr (Rio J).

[21] Mossey PA, Little J, Munger RG, Dixon MJ, Shaw WC (2009). Cleft lip and palate.. The Lancet.

[22] Molena KF, Winckler VPSV, Dalben GS (2021). Prevalence of cleft lip and palate in Bauru, SP – concordance among registries of HRAC/USP, DNV and SINASC.. Braz Dent Sci.

[23] Nunes LMN, Pereira AC, Queluz DP (2010). Fissuras orais e sua notificação no sistema de informação: análise da Declaração de Nascido Vivo (DNV) em Campos dos Goytacazes, Rio de Janeiro, 1999-2004.. Cien Saude Colet.

[24] Santana TM, Silva MDP, Brandão SR, Gomes AOC, Pereira RMR, Rodrigues M (2015). Nascidos vivos com fissura de lábio e/ou palato: as contribuições da fonoaudiologia para o Sinasc.. Revista CEFAC.

