# Supplementary material for: Underreporting of oral cleft cases: a descriptive study, western Paraná, 2015-2019
Source: Epidemiol Serv Saude. 2026 Mar 20;35:e20240716. doi: 10.1590/S2237-96222026v35e20240716.en (PMC13008300; doi:10.1590/S2237-96222026v35e20240716.en)
Supplement: Translation

# Translation: Subnotificação de casos de fissuras orais: estudo descritivo, oeste do Paraná, 2015-2019

**DOI:** 10.1590/S2237-96222026v35e20240716.pt

**Authors:** Isabelle Leticia Bender de Souza, Andressa Brum, Aline Nardelli, Mariângela Monteiro de Melo Baltazar, Fabiana Gonçalves de Oliveira Azevedo Matos, Luciana Paula Grégio d’Arce

**Affiliations:** 1 Universidade Estadual do Oeste do Paraná, Cascavel, PR, Brasil

**Keywords:** Anomalias Congênitas, Fissura Palatina, Fenda Labial, Serviço de Saúde, Epidemiologia

## Abstract

**Objetivos:**

Verificar se houve subnotificação de casos de fissuras orais no Sistema de Informações Sobre Nascidos Vivos (Sinasc) na 10ª Regional de Saúde do Paraná, analisando a concordância dos números com os registrados em um centro de referência na mesma região, bem como definir a prevalência média no período 2015-2019.

**Métodos:**

Trata-se de estudo epidemiológico, descritivo e retrospectivo. Os dados foram obtidos a partir da 10ª revisão da Classificação Internacional de Doenças de ambos os bancos de dados. Utilizou-se como medida de frequência a razão entre casos e nascidos vivos no mesmo ano e local.

**Resultados:**

Foram analisados 121 casos, sendo 44 casos com base nos dados do Sinasc e 77 com base nos dados do centro de referência. Segundo o Sinasc, houve prevalência de 8,75 casos a cada 10 mil habitantes. Conforme o centro de referência, houve prevalência de 16,87 casos a cada 10 mil habitantes, o que indicou a subnotificação de 42,86%. Com relação à análise da classificação das fissuras orais, 68,19% dos casos tiveram seu tipo de fissura determinado no Sinasc.

**Conclusão:**

O estudo demonstrou que, na 10ª Regional de Saúde do Paraná, houve subnotificação no Sinasc das pessoas nascidas com fissura oral entre 2015 e 2019. Isso talvez pudesse ter sido resolvido, caso houvesse edição dos dados da declaração de nascidos vivos após o nascimento quando a fissura fosse detectada.

**Aspectos éticos**

Esta pesquisa respeitou os princípios éticos, obtendo os seguintes dados de aprovação:

Comitê de ética em pesquisa: Universidade Estadual do Oeste do Paraná

Número do parecer: 4.250.143

Data de aprovação: 1/9/2020

Certificado de apresentação de apreciação ética: 36452320.0.0000.0107

Registro do consentimento livre e esclarecido: Não se aplica.

## Introdução 

As fissuras orais são as anomalias congênitas mais frequentes na população entre as craniofaciais (1-3). A etiologia das fissuras orais está relacionada a fatores hereditários, dados estimados entre 25% e 30% em 2023, podendo ser relacionadas a herança mendeliana ou a alterações cromossômicas (4), e a uma gama de fatores ambientais multifatoriais. Esses fatores incluem deficiências nutricionais, alcoolismo, estilo de vida e exposição dos pais a substâncias químicas, como agrotóxicos, correspondendo entre 70% e 80% das ocorrências em 2019 (5,6). Também se relacionaram sazonalidade, grupo étnico, idade dos pais, peso ao nascer e classe social à etiologia das fissuras orais (1,7-9). 

A prevalência de nascidos vivos com fissuras orais no Brasil foi de aproximadamente 5,2 a cada 10 mil nascimentos em 2022 (10). Em 2025, a região Sul continuou destacando-se com a maior prevalência de fissuras no país (5,10,11). As malformações craniofaciais, como as fissuras orais, apresentam tendência ascendente e de grande importância epidemiológica, além de impactos sociais e econômicos para o indivíduo, sua família e a sociedade em geral (5,12). O tratamento necessita de abordagem integrada com equipe multiprofissional altamente especializada (13).

Devido à necessidade de monitoramento de dados em saúde, o Sistema Único de Saúde implementou ferramentas como o Departamento de Informática do Sistema Único de Saúde (DATASUS), visando sistematizar e propagar informações através de transferência eletrônica de dados. Além disso, o DATASUS é o local de produção de dados de indicadores operacionais e epidemiológicos que fundamentam as políticas públicas em saúde (14,15). 

No DATASUS, concentram-se os dados do Sistema de Informações Sobre Nascidos Vivos (Sinasc), no qual o sistema de 10ª revisão da Classificação Internacional de Doenças (CID-10) é utilizado para classificar os diversos tipos de fissuras. Estes correspondem aos CID-10 Q35 (fissura palatina), Q36 (fissura labial) e Q37 (fissura labiopalatina) (16). 

Quando nasce um bebê, o profissional da saúde deve preencher a Declaração de Nascido Vivo, documento base para a entrada de dados no Sinasc. Ele contém as informações gerais do recém-nascido, e, no item 6, informa-se se foi detectado alguma anomalia congênita, a qual deve ser descrita no item 41. Neste espaço, coloca-se o CID-10 correspondente, inclusive as fissuras orais (17).

Este estudo teve como objetivo verificar se houve subnotificação dos casos de fissuras orais entre recém-nascidos entre 2015 e 2019 na 10ª Regional de Saúde do Paraná, a partir da comparação entre os dados registrados no Sinasc e os registrados em um centro de referência em fissuras orais presente na mesma região. Além disso, teve como objetivo determinar a prevalência de fissuras orais nessa população. 

## Métodos 

### Delineamento 

Trata-se de estudo epidemiológico, descritivo e retrospectivo, realizado entre março e julho de 2024. Analisaram-se prontuários físicos e eletrônicos do Centro de Atenção e Pesquisa em Anomalias Craniofaciais, localizado no Hospital Universitário do Oeste do Paraná, e dados do Sinasc entre 2015 e 2019.

### Contexto 

O estudo empregou dados obtidos referentes à 10ª Regional de Saúde do Paraná (região de Cascavel, Paraná) tanto do Sinasc quanto do centro de referência.

O referido centro existe desde 2013, sendo oficialmente credenciado ao Sistema Único de Saúde em 2018 para tratamento de alta complexidade de pessoas com fissuras labiopalatais. Oferece tratamento especializado para pessoas com anomalias craniofaciais, especialmente fissuras labiopalatinas, provenientes principalmente das regiões oeste e sudoeste do Paraná, além de outros estados, como Santa Catarina e Mato Grosso do Sul, e países fronteiriços, como Paraguai e Venezuela.

### Tamanho do estudo

A 10ª Regional de Saúde abrange 25 municípios da região oeste do estado. A população foi estimada em 510 mil habitantes em 2025.

### Fonte de dados

Foram contabilizados todos os prontuários do centro de referência pertencentes à 10ª Regional de Saúde e os nascimentos registrados no Sinasc na mesma regional, entre 2015 e 2019, utilizando o município da mãe como o de residência, critério utilizado pelo Sinasc.

### Coleta de dados

Para a obtenção dos dados do Sinasc, foram utilizados como termos da CID-10:

- Q35 para os tipos de fenda palatinas (Q35.1 Fenda do palato duro; Q35.3 Fenda do palato mole; Q35.5 Fenda do palato duro com fenda do palato mole; Q35.7 Fenda da úvula; e Q35.9 Fenda palatina não especificada);
- Q36 para as fendas labiais (Q36.0 Fenda labial bilateral; Q36.1 Fenda labial mediana; e Q36.9 Fenda labial unilateral); e
- Q37 para fenda labial com fenda palatina (Q37.0 Fenda do palato duro com fenda labial bilateral; Q37.1 Fenda do palato duro com fenda labial unilateral; Q37.2 Fenda do palato mole com fenda labial bilateral; Q37.3 Fenda do palato mole com fenda labial unilateral; Q37.4 Fenda dos palatos duro e mole com fenda labial bilateral; Q37.5 Fenda dos palatos duro e mole com fenda labial unilateral; Q37.8 Fenda do palato com fenda labial bilateral, não especificada; e Q37.9 Fenda do palato com fenda labial unilateral, não especificada).

Os dados foram coletados manualmente do Sinasc, cruzando-se as informações por CID-10, e tabulados no programa Microsoft Excel. 

Os dados do centro foram obtidos a partir da análise de prontuários de pessoas que nasceram no referido período de análise, com base na descrição do tipo de fissura pela equipe multiprofissional do centro. 

### Métodos estatísticos

Para avaliar a sensibilidade do Sinasc na identificação de casos de fissuras orais, realizou-se a comparação com o centro de referência. Foram considerados como verdadeiros positivos os casos registrados simultaneamente nos dois sistemas e como falsos negativos os casos registrados exclusivamente pelo centro de referência. A sensibilidade foi calculada como a razão entre o número de casos registrados em ambos os sistemas e a soma dos verdadeiros positivos com os falsos negativos.

As prevalências de fissuras orais em recém-nascidos, tanto do centro de referência quanto do Sinasc, foram calculadas pela razão entre o número de nascidos vivos com fissuras pelo total de nascimentos presente no Sinasc, ambos no mesmo ano e local, multiplicado por 10 mil. A avaliação do tipo de fissura baseou-se nos dados do Sinasc, os quais as categorizam de acordo com a CID-10, em que as fissuras palatais foram designadas como Q35; as labiais, como Q36; e as labiopalatais, como Q37. As diferenças entre os conjuntos de dados foram calculadas, e as taxas percentuais relevantes foram estabelecidas. As fissuras sem CID-10 especificado não foram somadas para a análise por tipo de fissura. 

Para avaliar se houve diferença estatística entre os resultados dos bancos de dados, as variáveis foram analisadas quanto ao pressuposto de normalidade (teste de Shapiro-Wilk). Em casos de adequação ao pressuposto, as comparações entre as instituições foram realizadas por meio do teste T para amostras dependentes unilateral e, quando contrário, foi empregado o teste não paramétrico de Wilcoxon, com significância de 5%. Os mapas foram gerados através do software livre QGIS.

## Resultados 

Durante a análise do período em questão, registraram-se 44 casos de fissuras orais com base nos dados do Sinasc, enquanto o centro de referência notificou 77 casos. A sensibilidade do Sinasc foi 57,14%, o que indica que o sistema foi capaz de captar pouco mais da metade dos casos efetivamente registrados pelo centro de referência. 

A avaliação dos registros do Sinasc revelou a prevalência média de 8,75 casos de fissuras orais a cada 10 mil nascimentos no intervalo 2015-2019, o que correspondeu a 1 caso a cada 1.143 nascidos. A prevalência foi de 16,87 casos a cada 10 mil nascimentos segundo os dados do centro de referência, equivalentes a 1 caso a cada 593 nascimentos (Figura 1). A taxa de subnotificação foi 42,86%. Houve diferença estatisticamente significativa entre as médias de prevalência dos bancos de dados analisados (p-valor 0,001). 

**Figura 1:** Prevalência a cada 10 mil nascimentos do Sistema de Informações Sobre Nascidos Vivos (Sinasc) e do centro de referência na 10ª Regional de Saúde. Paraná, 2015-2019

De acordo com o Sinasc, a prevalência média dessas fissuras na 10ª Regional de Saúde do Paraná em 2015 foi de 8,9 casos a cada 10 mil habitantes; reduzindo para 2,5 em 2016 e para 1,5 em 2017; aumentando para 17,4 em 2018; e alcançando 13,4 em 2019. Para o centro de referência, a prevalência foi de 15,3 casos para cada 10 mil nascimentos em 2015; diminuindo para 12,5 em 2016 e para 5,8 em 2017; e aumentando expressivamente para 25,8 em 2018 e 24,2 em 2019. Ao comparar as prevalências, observou-se o aumento de casos de fissuras orais, especialmente em 2018 e 2019. A prevalência foi maior no centro de referência quando comparado ao Sinasc nos anos citados (t=-7,98; p-valor 0,001) (Figura 2).

**Figura 2:** Prevalência média anual de fissuras orais a cada 10 mil nascimentos do Sistema de Informações Sobre Nascidos Vivos (Sinasc) e do centro de referência na 10ª Regional de Saúde. Paraná, 2015-2019

Entre os casos do sexo masculino, observou-se diferença estatística entre as médias de prevalência do centro de referência em relação ao Sinasc (p-valor 0,011). A prevalência foi de 10,34 casos a cada 10 mil nascimentos conforme os dados do Sinasc, enquanto o centro de referência registrou 22,29 casos. Entre os casos do sexo feminino, não houve diferença estatística entre os bancos de dados (p-valor 0,421), com médias de 6,70 no Sinasc e 10,46 casos a cada 10 mil nascimentos no centro de referência.

Conforme os dados do Sinasc, 1 município apresentou prevalência entre 60 e 70 casos para 10 mil nascimentos, 3 municípios registraram prevalência entre 20 e 30 casos, e 4 municípios tiveram prevalência entre 10 e 20 casos (Figura 3A). Dezoito municípios apresentaram prevalência inferior a 10 casos a cada 10 mil habitantes (Figura 3A). 

**Figura 3:** Prevalência de fissuras orais a cada 10 mil nascimentos entre 2015 e 2019 por município da 10ª Regional de Saúde do Paraná, segundo os dados do: (A) Sistema de Informações Sobre Nascidos Vivos; (B) centro de referência, 2015-2019

Segundo os dados do centro de referência, 1 município registrou prevalência entre 60 e 70 casos para 10 mil nascimentos, 4 municípios apresentaram prevalência entre 40 e 50 casos, 1 município teve prevalência entre 30 e 40 casos, 2 municípios registraram prevalência entre 20 e 30 casos, e 6 municípios apresentaram prevalência entre 10 e 20 casos (Figura 3B). Onze municípios apresentaram prevalência abaixo de 10 casos para cada 10 mil nascimentos (Figura 3B).

Os casos de fissuras foram classificados de maneira simplificada em labial, palatal e labiopalatal. Analisando os dados do Sinasc sem distinção por sexo, a fissura palatal representou 8 casos (18,9%), sendo a menos frequente, seguida das fissuras labiais com 9 casos (20,5%) e labiopalatais com 13 casos (29,6%). Catorze ocorrências de fissuras registradas no Sinasc não apresentavam CID especificada no sistema (31,8%). O tipo de fissura foi determinado em 68,2% dos casos.

Os dados do centro de referência indicaram que tanto as fissuras palatais quanto as labiopalatais compreenderam 29 casos (37,7%) do total. As fissuras labiais somaram 19 casos (24,7%) (Figura 4). Entre os casos de fissura labial registrados pelo centro de referência, 52,7% não foram especificados no Sinasc. Para as fissuras labiopalatais, esse número foi 55,2%, sendo a maior discrepância observada nas fissuras palatais, as quais atingiram 72,4%.

**Figura 4:** Número de casos por tipo simplificado de fissura registrados no Sistema de Informações Sobre Nascidos Vivos (Sinasc) e no centro de referência da 10ª Regional de Saúde. Paraná, 2015-2019

## Discussão 

Este estudo analisou os dados sobre fissuras orais do Sinasc, comparando-os com os de um centro de referência. Constatou-se prevalência maior das fissuras orais na 10ª Regional de Saúde em relação à média paranaense e identificou-se subnotificação das fissuras inseridas no Sinasc. 

A prevalência de casos de fissuras orais conforme os registros do Sinasc e do centro de referência, respectivamente de 8,75 e de 16,87 a cada 10 mil habitantes, mostrou-se superior à de estudos nacionais anteriores. No estado de São Paulo, entre 1975 e 1994 na região, observou-se prevalência de 1,9 caso a cada 10 mil nascimentos (18). Dados a partir de 2000 apontaram prevalências maiores: 5,8 no Rio Grande do Norte em 2017 (19), 5,2 em Minas Gerais em 2022 (10) e 5,1 casos no Paraná em 2019 (5) a cada 10 mil habitantes.

A região Sul detém as maiores taxas de fissuras orais no país, com prevalência em 2022 de 7,16 casos para 10 mil nascimentos (10), 7,9 casos em 2019 (5), e 8,23 casos em 2017 (19). Comparando-se as prevalências da literatura com as descritas neste estudo, notou-se que a 10ª Regional de Saúde do Paraná apresentou prevalências mais elevadas tanto nos dados obtidos do Sinasc como também nos obtidos do centro de referência.

A prevalência de fissuras orais aqui relatadas para o centro de referência, para uma única regional de saúde, foi maior que a relatada anteriormente para o Paraná (6,8 casos a cada 10 mil nascimentos) (19). Do mesmo modo, outro trabalho publicado com dados de outro centro de especializado em fissuras orais estimou essa prevalência em 9,9 casos a cada 10 mil nascimentos, também maior que a média do Paraná (20). 

Esses valores superiores identificados na prevalência de fissuras orais em centros de referências destacam a importância de uma investigação mais aprofundada para compreender os fatores específicos que contribuem para essas variações regionais nas taxas de fissurados. Além disso, contribuem para estratégias mais eficazes de prevenção e intervenção em casos de fissuras orais.

Quanto aos dados de prevalência de fissuras orais encontrados na literatura, eles diferem entre si devido a vários fatores, incluindo etiologias multifatoriais e dificuldade em obter e caracterizar amostras com precisão. Esses fatores explicam, em parte, as inconsistências entre as bases de dados públicas e os centros de referências (21,22). 

O Sinasc é alimentado com os dados das declarações de nascidos vivos, visando coletar informações sobre as gestantes e os recém-nascidos, com o objetivo de delinear um perfil epidemiológico. Embora as declarações incluam campos destinados às informações sobre anomalias congênitas, a subnotificação ocorre frequentemente devido a diversas razões, como a falta de diagnóstico ao nascer e o desconhecimento da necessidade de notificação por parte dos profissionais que preenchem o documento. A limitação do prazo de inserção no sistema compromete a qualidade dos dados e dificulta o planejamento de ações voltadas à atenção neonatal e ao cuidado especializado.

Os casos de fissuras orais, quando não notificados na declaração de nascidos vivos, serão incluídos posteriormente nos bancos de dados públicos (como números relativos) após o início dos atendimentos realizados no sistema público de saúde (geralmente em centros de referência especializados). Mesmo com defasagem temporal, tais centros desempenham papel significativo na coleta de dados epidemiológicos, apresentando maior precisão na apuração da totalidade de casos. 

Este estudo identificou a taxa de subnotificação de 43%, sendo as fissuras palatais as mais subnotificadas, atingindo 72%. No estado do Rio de Janeiro, foi identificada a taxa de subnotificação de 45% nas declarações de nascidos vivos quanto à presença de fissuras orais, sendo as fissuras palatais as mais subnotificadas, com 65% (23).

A constatação dessa subnotificação neste trabalho também está alinhada com descobertas prévias apontadas na literatura (20,22,24). Essa complexidade na obtenção e integração de dados reforça a necessidade de aprimoramento nos sistemas de notificação e registro para uma avaliação mais precisa da prevalência de fissuras.

A predominância da subnotificação entre as fissuras palatais pode ser explicada pela dificuldade em visualizá-las imediatamente após o nascimento, muitas vezes requerendo dias ou até meses para serem identificadas. Um dado adicional de preocupação é que 68% dos casos têm o tipo de fissura determinado no Sinasc. Essas informações não apenas são cruciais para justificar repasses de verbas, mas também são fundamentais para a definição de políticas públicas eficazes na regional de saúde. Mesmo o centro de referência analisado neste estudo sendo incorporado à rede Sistema Único de Saúde apenas em 2018 (apesar de atuante desde 2013), é evidente que aprimorou a coleta de novos dados, bem como dos retroativos.

A discrepância entre os bancos de dados ressalta a necessidade de aprimoramento na coleta dos dados que alimentam o Sinasc. Uma estratégia seria a melhor capacitação dos profissionais responsáveis pelo preenchimento da declaração de nascidos vivos, dado o papel crucial desempenhado por eles na delimitação de perfis epidemiológicos e na implementação de centros especializados e de referência para o tratamento integrativo dessas pessoas, além do planejamento de repasses de verbas coerentes com a realidade.

Outra estratégia viável para aumentar a confiabilidade dos dados seria a possibilidade de inserção posterior ou modificação deles na plataforma do Sinasc. Essa abordagem flexível poderia oferecer uma maneira mais dinâmica de atualizar e corrigir informações, contribuindo assim para uma base de dados mais precisa e útil para a formulação de políticas de saúde eficazes.

A 10ª Regional de Saúde do Paraná apresentou uma prevalência de casos de fissura superior àquela previamente identificada para o Paraná, para a região Sul e até mesmo para a prevalência nacional. Apesar de este estudo focar uma regional de saúde específica, acredita-se que ele reflita uma realidade mais ampla de subnotificação de dados públicos em saúde em nível nacional. Esse problema requer discussão mais abrangente, a fim de identificar maneiras de minimizar essa discrepância e mitigar seus efeitos. A importância de abordar a subnotificação é fundamental para garantir a compreensão precisa da incidência de fissuras orais e, por conseguinte, desenvolver estratégias eficazes de intervenção.

## Data Availability

O banco de dados está disponível em: http://doi.org/10.17632/n9pvg96h55.1.
